# Predictors of persisting pain in children with Juvenile Idiopathic Arthritis: a case control study nested in the ReACCh-Out cohort

**DOI:** 10.1186/s12969-023-00885-w

**Published:** 2023-09-16

**Authors:** Tara McGrath, Jaime Guzman, Lori Tucker, Natalie J. Shiff, Maryna Yaskina, Susan Tupper, Dax G. Rumsey, for the Research in Arthritis in Canadian Children Emphasizing Outcomes (ReACCh-Out) Investigators, Susanne Benseler, Roberta Berard, Gilles Boire, Roxana Bolaria, David Cabral, Bonnie Cameron, Sarah Campillo, Mercedes Chan, Gaëlle Chédeville, Anne-Laure Chetaille, Paul Dancey, Jean Dorval, Ciarán Duffy, Janet Ellsworth, Brian Feldman, Debbie Feldman, Katherine Gross, Ellie Haddad, Kristin Houghton, Adam Huber, Nicole Johnson, Roman Jurencak, Bianca Lang, Maggie Larché, Ronald Laxer, Claire LeBlanc, Deborah Levy, Nadia Luca, Paivi Miettunen, Kimberly Morishita, Kiem Oen, Ross Petty, Suzanne Ramsey, Alan Rosenberg, Johannes Roth, Claire Saint-Cyr, Heinrike Schmeling, Rayfel Schneider, Earl Silverman, Lynn Spiegel, Elizabeth Stringer, Rosie Scuccimarri, Shirley Tse, Stuart Turvey, Karen Watanabe Duffy, Rae Yeung

**Affiliations:** 1 Department of Pediatrics, Division of Rheumatology, University of Alberta, K3-508 ECHA; 11405 87 Ave NW, Edmonton, AB T6G 1C9 Canada; 2 Department of Pediatrics, Division of Rheumatology, University of British Columbia, Vancouver, Canada; 3 Adjunct, Department of Community Health and Epidemiology, University of Saskatchewan and Janssen Scientific Affairs LLC, Horsham, PA UK; 4 Women’s and Children’s Health Research Institute, University of Alberta, Edmonton, Canada; 5 Department of Pediatrics, University of Saskatchewan, Saskatoon, Canada

**Keywords:** Pain, Juvenile idiopathic arthritis, Enthesitis, Quality of life, Children

## Abstract

**Background:**

To identify baseline predictors of persisting pain in children with Juvenile Idiopathic Arthritis (JIA), relative to patients with JIA who had similar baseline levels of pain but in whom the pain did not persist.

**Methods:**

We used data from the Research in Arthritis in Canadian Children emphasizing Outcomes (ReACCh-Out) inception cohort to compare cases of ‘moderate persisting pain’ with controls of ‘moderate decreasing pain’. Moderate pain was defined as a Visual Analogue Scale (VAS) for pain measurement score of > 3.5 cm. Follow-up was minimum 3 years. Univariate and Multivariate logistic regression models ascertained baseline predictors of persisting pain.

**Results:**

A total of 31 cases and 118 controls were included. Mean pain scores at baseline were 6.4 (SD 1.6) for cases and 5.9 (1.5) for controls. A greater proportion of cases than controls were females (77.4% vs 65.0%) with rheumatoid factor positive polyarthritis (12.9% vs 4.2%) or undifferentiated JIA (22.6% vs 8.5%). Oligoarthritis was less frequent in cases than controls (9.7% vs 33%). At baseline, cases had more active joints (mean of 11.4 vs 7.7) and more sites of enthesitis (4.6 vs 0.7) than controls. In the final multivariate regression model, enthesitis count at baseline (OR 1.40, CI 95% 1.19–1.76), female sex (4.14, 1.33–16.83), and the overall Quality of My Life (QoML) baseline score (0.82, 0.69–0.98) predicted development of persisting pain.

**Conclusions:**

Among newly diagnosed children with JIA with moderate pain, female sex, lower overall quality of life, and higher enthesitis counts at baseline predicted development of persisting pain. If our findings are confirmed, patients with these characteristics may be candidates for interventions to prevent development of chronic pain.

## Background

Pain in Juvenile Idiopathic Arthritis (JIA) has been described as the most distressing component of the disease [1]. It may persist despite treatment, even in those whose arthritis has resolved [2]. Identifying predictors of persisting pain early in the course of the disease may enable better targeting of pain management interventions and hopefully prevent the development of persisting pain.

Two recent studies in children with JIA that used latent trajectory analysis [3] or group-based trajectory analysis [4] identified an uncommon persisting pain trajectory over 5 years. Shiff et al. referred to this as “chronically moderate pain” and reported its occurrence in 7.4% of patients [3]. Rashid et al. reported finding “consistently high pain” in 17.3% of JIA patients studied [4]. Both studies found many baseline clinical and demographic characteristics distinguishing these patients from those with minimal or low pain at baseline, but few characteristics could distinguish individuals in this “chronic, moderate, or high pain” group from patients who started with similar levels of pain but whose pain did not persist. The only baseline characteristics significantly associated with persisting pain were older age in the study by Shiff et al., and older age and higher baseline disability in the study by Rashid et al. [3, 4]. Although having Enthesitis-Related Arthritis (ERA), undifferentiated arthritis, or enthesitis regardless of JIA category have been associated with worse pain outcomes in other studies [5, 6], neither JIA category nor enthesitis were identified in these two studies as predictors of persisting pain.

Both Shiff et al. and Rashid et al. used probabilistic assignment of participants (i.e., each patient was estimated to contribute in different degrees to one or more trajectories, even if pain was measured only once or twice) [3, 4]. It is unclear whether this probabilistic assignment could be a factor contributing to the difficulty in distinguishing patients that develop persisting pain from those in whom pain decreased. We hypothesized that contrasting patients who clearly had documented persisting pain with those who clearly had documented decreasing pain in a case control study might be more fruitful.

We conducted a case–control study to identify baseline clinical or demographic predictors that distinguish children with JIA whose initial moderate pain becomes persistent from those whose initial moderate pain does not persist. Identifying predictors of persistent pain early in the course of disease may enable health care providers to target treatment and management interventions earlier. This is a key initial step in the prevention of pain 'chronification' in young people with JIA and has the potential for substantial, long term positive impact at the individual, institutional, and societal levels [7].

## Methods

The Research in Arthritis in Canadian Children emphasizing Outcomes (ReACCh-Out) study methods have previously been reported [8]. In brief, children newly diagnosed with JIA were recruited at 16 Canadian centres between 2005 and 2010 and followed for up to 5 years. Patients were followed every 6 months for two years and then annually for up to 5 years. At each follow-up visit, pediatric rheumatologists reported clinical data and patients/parents completed questionnaires.

In the current study, cases were defined as children with JIA followed for a minimum of 3 years, who had at least moderate pain (VAS pain score > 3.5 cm) at baseline, at their final visit, and at least one interval visit [9]. Controls were defined as children with JIA followed for a minimum of 3 years, who had a VAS pain score > 3.5 cm at baseline that decreased to ≤ 3.5 cm from 12 months after diagnosis until the end of follow-up. This definition was chosen as it has been shown that most children with JIA on effective treatment attain inactive disease after approximately one year [10].

Arthritis-related pain intensity in the past week was measured using a validated 10 cm horizontal VAS (0 = no pain, 10 = very severe pain). Parents were instructed to obtain the pain score from their child if possible or to have the child complete the questionnaires themselves if they had the capacity to do so. Otherwise, parents provided pain scores on behalf of their child. Other patient-reported outcome measures included the Juvenile Arthritis Quality of Life Questionnaire (JAQQ, from 1 = best to 7 = worst) [11], the Quality of My Life questionnaire overall scale (QoML, 0 = worst, 10 = best) [12], the Childhood Health Assessment Questionnaire disability index (CHAQ, 0 = best, 3 = worst) [13] and the patient/parent global assessment (PPA; 0 = best, 10 = worst) [11]. These patient/parent-reported outcomes (PROs) are validated outcome measures well studied in the JIA population. The JAQQ measures health-related quality of life in children with JIA according to difficulties experienced in 4 domains (gross motor, fine motor, psychosocial, symptoms), while the QoML is a global self-reported quality of life scale [11, 12]. The CHAQ assesses physical disability in children with JIA and is an adaptation of the Stanford Health Assessment Questionnaire [13].

Physician assessments included the number of active joints and enthesitis sites documented via 71-joint and 33-enthesitis homunculi. JIA category was assigned by the treating rheumatologist based on the ILAR criteria and confirmed via ReACCh-Out investigators [14]. Among multiple demographic variables collected at baseline in ReACCH-Out only age and sex were included in our study as there is literature supporting specific influence of these variables on the JIA pain experience and trajectory [3, 4, 15, 16].

### Statistical analyses

All statistical analyses were performed using SAS version 9.4 (SAS Institute Inc., Cary, NC, USA). Continuous data were summarized using means and standard deviations (SD), and categorical data with absolute and relative frequencies (n and %). Univariate binomial logistic regression was used to calculate odds ratios (OR) and 95% CI for the association of candidate baseline predictors with persisting pain. Among the data available in the ReACCh-Out cohort, the authors selected a limited set of candidate predictors based on previously reported associations [3–6], and the biopsychosocial model for chronic pain [17]. We were cautious to avoid the inclusion of too many variables in prediction models, relative to number of available cases. The predictors assessed were age at JIA onset, sex, JIA category, enthesitis count at baseline, active joint count at baseline, CHAQ, QoML, JAQQ, JAQQ psychosocial domain, and PPA. JAQQ item 14 “felt depressed” and item 16 “felt sad” were considered individually in exploratory analyses, as there is evidence that patients with JIA and depressive symptoms experience more pain, and no validated depression scales were available in the ReACCh-Out dataset [18]. Variables with p ≤ 0.2 from the univariate logistic regression were considered for the multivariate logistic regression model.

## Results

**Fig. 1:**
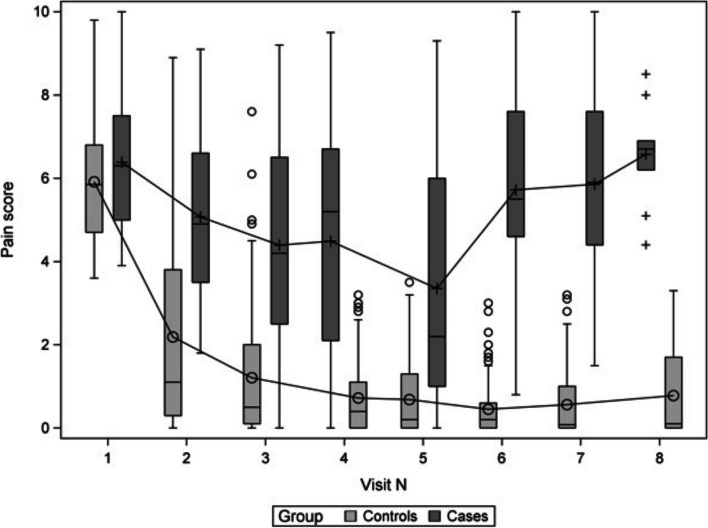
Box Plot of Pain Scores Over Time in Children with Persisting Pain (Cases) Versus those with Decreasing Pain (Controls). The length of the box represents the interquartile range (the distance between the 25th and 75th percentiles). The circle within the box represents the control group mean and the cross within the box represents the case group mean. The horizontal line within the box represents the group median. The vertical lines, or ‘whiskers’, are drawn to the most extreme points in the group that lie within the fences. The upper fence is defined as the third quartile (represented by the upper edge of the box) plus 1.5 times the interquartile range (IQR). The lower fence is defined as the first quartile (represented by the lower edge of the box) minus 1.5 times the interquartile range. Visit *N* = visit number. Visit 1 = initial visit, visit 2 = 6-month visit, visit 3 = 12-month visit, visit 4 = 18-month visit, visit 5 = 2-year visit, visit 6 = 3-year visit, visit 7 = 4-year visit, visit 8 = 5-year visit

Among 1497 children with JIA enrolled in the ReaCCh-Out cohort, 663 were followed for at least 3 years. Among these 663 participants, 227 (34.2%) of those had a pain VAS > 3.5 cm at baseline and 31 (4.7%) met criteria as cases and 118 (17.8%) as controls for the current study. Some potential cases and controls did not meet the inclusion criteria due to missing pain scores. Baseline characteristics of cases and controls are shown in Table 1; and pain scores over time for cases versus controls are shown in Fig. 1.

**Table 1:** Baseline characteristics of cases and controls

		**Cases: Moderate persisting pain**	**Controls: Moderate decreasing pain**
**Patients, n**		31	118
**Females, n (%)**		24 (77.4)	76 (64.4)
**JIA Category, n (%)**	**Oligoarthritis**	3 (9.7)	36 (30.5)
	**RF -ve Polyarthritis**	6 (19.4)	27 (22.9)
	**RF +ve Polyarthritis**	4 (12.9)	5 (4.2)
	**Enthesitis-Related Arthritis**	7 (22.6)	23 (19.5)
	**Psoriatic**	3 (9.7)	8 (6.8)
	**Systemic**	1 (3.2)	9 (7.6)
	**Undifferentiated**	7 (22.6)	10 (8.5)
**Mean Active Joint Count at Baseline, mean (SD)**		11.42 (12.15)	7.66 (10.03)
**Mean Enthesitis Count at Baseline, mean (SD)**		4.63 (6.25)	0.66 (1.77)
**Mean Age at Onset of JIA, mean (SD)**		9.06 (4.22)	7.63 (4.65)
**Mean Pain Score at Baseline, mean (SD)**		6.39 (1.65)	5.92 (1.47)

A greater proportion of cases than controls were female (77.4% vs 65.0%) with rheumatoid factor positive polyarthritis (12.9% vs 4.2%) or undifferentiated JIA (22.6% vs 8.5%). Oligoarthritis was less frequent in cases (9.7% vs 33%). Cases had a higher mean active joint count (11.4 vs 7.7) and more sites of enthesitis (4.6 vs 0.7) at baseline. A baseline enthesitis count was not entered in 64 patients: in 48 of these, it was stated that enthesitis was not applicable. Since none of these patients had any sites of enthesitis marked on the homunculus, a value of zero was assumed.

Baseline predictors associated with persisting pain are shown in Table 2.

**Table 2:** Baseline predictors of persisting pain in children with juvenile arthritis

Variables (at baseline)		Univariate model Odds ratio (95% CI)	*P*-value	Final model Odds ratio (95% CI)	*P*-value
Age at onset (years)		1.07 (0.98—1.18)	0.14		
Sex (female vs male)		1.85 (0.77—4.98)	0.19	4.14 (1.33—16.83)	0.03*
JIA Category	Oligoarthritis	reference	0.09		
	Enthesitis-Related Arthritis	3.54 (2.03—6.34)			
	Systemic	4.23 (2.13—8.42)			
	Psoriatic	2.96 (1.71—5.09)			
	RF -ve Polyarthritis	1.58 (0.63—3.64)			
	RF +ve Polyarthritis	10.88 (5.54—21.90)			
	Undifferentiated	8.26 (4.67—15.12)			
Enthesitis Count (each additional site)		1.30 (1.12—1.56)	0.002*	1.40 (1.19—1.76)	0.0005*
Active Joint Count (each additional joint)		1.03 (1.00—1.07)	0.08		
CHAQ score		1.22 (0.66—2.21)	0.52		
QoML score		0.85 (0.72—0.99)	0.03*	0.82 (0.69—0.98)^a^	0.02*
Patient/Parent global assessment score		1.25 (1.06—1.48)	0.01*	1.07 (0.88—1.31)	0.49
JAQQ total score		1.08 (0.75—1.54)	0.69		
JAQQ psychosocial score		1.08 (0.81—1.43)	0.58		
JAQQ ‘depression’ item		1.17 (0.91—1.50)	0.21		
JAQQ ‘sad’ item		1.00 (0.87—1.00)	0.85		

*CHAQ* Childhood Health Assessment Questionnaire, *QoML* Quality of My Life (overall score), *JAQQ* Juvenile Arthritis Quality of Life Questionnaire

*Denotes statistical significance

^a^For QoML score lower value is worse quality of life

A higher enthesitis count (OR 1.30, CI 95% 1.12–1.56, *p* = 0.002), lower quality of life as measured by QoML (0.85, 0.72–0.99, *p* = 0.03), and higher PPA at baseline (1.25, 1.06–1.48, *p* = 0.01) were all statistically associated with a child having moderate persisting pain in univariate analyses. In multivariate logistic regression with baseline QoML score, PPA, enthesitis count, and sex as covariates, PPA was no longer statistically significant and was therefore removed from the final model. In the final, parsimonious multivariate logistic regression model, a higher baseline enthesitis count (OR 1.40, CI 95% 1.19–1.76; *p* = 0.0005), female sex (4.14, 1.33–16.83; *p* = 0.03), and lower baseline QoML (0.82, 0.69–0.98, *p* = 0.02) were statistically significant predictors that distinguished cases (persisting pain) from controls (decreasing pain).

## Discussion

This case–control study assessed baseline variables that distinguish children with JIA with persisting moderate pain from those whose pain improved despite similar levels of baseline pain. In a final parsimonious model, we found that female sex, higher number of enthesitis sites and lower QoML at baseline were significantly associated with persisting pain.

The association of female sex with persisting musculoskeletal pain has been previously reported [19]. Higher pain levels have also been associated with female sex in JIA specifically [15, 16]. Interestingly, Shiff’s study found that although female sex clearly distinguished trajectories of persisting moderate pain from minimal pain, it did not distinguish persisting from improving pain [3].Similarly, Rashid et al. did not find that female sex was a factor contributing to persistence of pain [4]. Of note, whereas both Shiff and Rashid found older age at JIA onset was a factor in the development of persistent pain, age did not emerge as a significant factor in our study. The reason for this variation is yet unclear and underscores the importance of future research into the role of age as a predictor of persistent pain in JIA.

Baseline enthesitis count was the strongest predictor of persisting pain in our study. Shiff et al. found no association with presence of enthesitis but did not assess the enthesitis count (i.e. the number of entheseal sites that were tender on exam) [3]. Rashid et al. did not assess enthesitis per se but noted increased frequency of ERA in children with persisting pain [4]. Support for the deleterious effects of enthesitis on pain intensity and quality of life for children with JIA has been shown in previous studies and this factor should be included in future investigations [5, 6]. We hypothesize that more widespread and severe disease (with more diffuse enthesitis) may have more impact on central mechanisms that maintain pain. It is also possible that mechanisms underlying prolonged pain, including central sensitization and lower pain pressure threshold, may lead to allodynia and hyperalgesia [20]. This may result in a falsely elevated enthesitis site count found by the detecting clinician.

The association of poor patient-reported outcomes (i.e. poorer self or parent assessed overall health status and quality of life and others) with higher pain intensity in children with JIA has been reported in several studies [4, 21–23]. Given that both cases and controls started with similar levels of pain, it is possible that a lower self-perceived quality of life at similar pain intensities is acting as an indicator of distress or catastrophizing, but we did not have validated measure of distress or catastrophizing to investigate directly. In the literature, quality of life appears to be a broad and individualized construct, only partially affected by health status [12]. Seid et al. demonstrated that multiple non-medical factors contribute to a child’s quality of life scores, including self-efficacy, coping, barriers to adherence, social support, parental distress, and access to care [24]. These factors were not measured in this study, therefore it is difficult to ascertain the reasons for lower baseline quality of life in the cases versus the controls. We suspect each case’s unique biological, psychological, and social experiences contributed to varying degrees. Future studies more broadly examining the role of these additional factors in the development of persistent pain in JIA would be extremely pertinent and valuable.

Major strengths of this study include the longitudinal data collection, the documented, rather than probability assigned pain trajectory, the use of validated measures of pain and other PROs, and the national scope of the study. Our study has several limitations. First, the number of cases was small, due in part to missing pain score data. Nonetheless, it was telling that despite ReACCh-Out being the largest prospective cohort of children with JIA when first published in 2015, we could only confirm the presence of moderate persisting pain in 31 (< 5%) of eligible subjects. Second, clinical assessment of enthesitis is subjective and there may be non-JIA reasons for tenderness over entheseal locations. Third, the pain VAS in our study could be completed by either the child or the parent. Considering that previous research found just 71% agreement between child and parent on this question, the potential for overestimation or underestimation of the child’s pain score must be considered [25]. Fourth, we could only study the potential predictors that were measured in the ReACCh-Out cohort. Factors that influence a child’s pain experience (such as anxiety, depressive symptoms, pain catastrophizing, pain interference, and parental distress) were not assessed in this cohort. Lastly, genetic, epigenetic, physical, and social environmental factors that could influence neurobiological mechanisms that contribute to pain persistence were also not assessed [7].

## Conclusions

In conclusion, this study found that among children with JIA who had moderate pain at baseline, female sex, lower overall quality of life, and higher number of enthesitis sites at baseline were associated with the development of persisting pain. Further research is needed to support these preliminary findings. If our findings are confirmed, children with these characteristics may benefit from a pain management focus early in their course of treatment. Timely implementation strategies to prevent pain ‘chronification’ in those at risk may ease the burden of chronic pain in children with JIA, which may ultimately have lifelong, far-reaching benefits.

## Data Availability

The datasets used and analysed during the current study are available from the corresponding author on reasonable request.

## Abbreviations

CHAQ: Childhood Health Assessment Questionnaire
CI: Confidence Interval
ERA: Enthesitis-Related Arthritis
ILAR: International League of Associations for Rheumatology
JAQQ: Juvenile Arthritis Quality of Life Questionnaire
JIA: Juvenile Idiopathic Arthritis
OR: Odds Ratio
PPA: Patient/Parent Global Assessment
PROs: Patient-Reported Outcomes
QoML: Quality of My Life Questionnaire
ReACCh-Out: Research in Canadian Children Emphasizing Outcomes
RF: Rheumatoid Factor
SAS: Statistical Analysis System
SD: Standard Deviation
VAS: Visual Analogue Scale
